# Correction: Novel ensemble learning approach with SVM-imputed ADASYN features for enhanced cervical cancer prediction

**DOI:** 10.1371/journal.pone.0298980

**Published:** 2024-02-12

**Authors:** Raafat M. Munshi

There are errors in the Funding section. The correct Funding statement is: This research work was funded by Institutional Fund Projects under grant no. (IFPIP: 1446-415-1443). The author gratefully acknowledges the technical and financial support provided by the Ministry of Education and King Abdulaziz University, DSR. Jeddah, Saudi Arabia.
