# Supplementary material for: Novel ensemble learning approach with SVM-imputed ADASYN features for enhanced cervical cancer prediction
Source: PLoS One. 2024 Jan 10;19(1):e0296107. doi: 10.1371/journal.pone.0296107 (PMC10781159; doi:10.1371/journal.pone.0296107)
Supplement: Attachment — Submitted filename: PlosONE_Reply_to_reviewer.pdf [file pone.0296107.s002.pdf]

Manuscript ID: PONE-D-23-24373

Article title: Novel Ensemble Learning Approach with SVM-Imputed ADASYN Features for Enhanced Cervical Cancer Prediction

November 23, 2023

Dear Academic Editor,

First of all, we would like to extend our sincere gratitude to Editor in Chief, Academic Editor, for allowing a re-submission of our manuscript, with an opportunity to address the reviewer's comments. We greatly appreciate the Reviewers' time spent reviewing the paper and assisting in its improvement. These comments are very helpful for revising and improving the paper, as well as the important guiding significance to the other research. We have studied the comments very carefully and made the corrections that we hope to meet with the final approval. We have done our best to modify the paper to reflect all the comments made by anonymous reviewers. These changes are fully addressed in this reply letter.

Below, we provide point-to-point responses explaining how we have addressed each of the Reviewer's comments. For clarity, our responses have been written in blue color. whenever we copy text from the manuscript, it is given in red color.

REVIEWER 1

Concern 1

Introduction section need revision, it is recommended to present one idea in one paragraph.

Response: We are thankful for your insightful comment. Respected reviewer, We have revised the introduction section.

Concern 2

Add some recently published works for cervical cancer survival prediction in related work section.

Response: The authors are grateful for your valuable suggestions. We have added recent papers in the related work section.

Concern 3

Research gap is not clear.

Response: We are thankful for your insightful comment. Respected reviewer, We have added a research gap in the paper with the following lines:

While recent studies have made significant strides in applying machine learning techniques, such as ensemble models and advanced sampling methods, to predict cervical cancer, there remains a need to comprehensively assess the combined impact of these approaches on improving accuracy, handling missing data,

addressing class imbalances, and extracting complex features. Moreover, limited research has specifically evaluated the effectiveness of integrating Convolutional Neural Networks in conjunction with traditional ML methods for cervical cancer prediction.

Concern 4

What is the motivation and rationale of the study.

Response: The authors highly appreciate your valuable suggestions. Respected reviewer, The motivation and rationale for this study stem from the pressing need within the medical field to enhance the accuracy and efficiency of cervical cancer diagnosis. Existing research has showcased the potential of machine learning techniques, yet there's a critical gap in comprehensively evaluating the combined impact of various methodologies, such as ensemble models, oversampling techniques, and neural network approaches. This study aims to fill this void by proposing a holistic framework that integrates these methods, intending to significantly improve prediction accuracy, handle data complexities, and address imbalances, thereby advancing the field of computer-aided diagnosis for cervical cancer.

Concern 5

Figure quality is not good.

Response: Respected reviewer, We have improved quality of figures.

Concern 6

Empirical analysis of the proposed methodology is missing.

Response: Respected reviewer, We have provided data sources, preprocessing steps, experimental setup, performance metrics, and results. Additionally, comparing the methodology's performance against existing approaches, and discussing the empirical findings, limitations, and potential improvements comprehensively address the empirical analysis.

Concern 7

Authors should re-write the Abstract based on novelty, Challenges, validation techniques, and some suggestion. Impact on social and Clinical.

Response: Respected reviewer, We have revised the abstract.

Concern 8

Stop using words like our work and we. Used proposed work or proposed model in the entire paper.

Response: We are thankful for your insightful comment. Respected reviewer, We have removed such words from the paper.

Concern 9

Stick to one term either F1 score or F1-score in the entire paper.

Response: Respected Reviewer, We have corrected it and used F1 score in the paper.

Concern 10

Finally, grammatical proofing also required to improve the interest of the readers.

Response: Respected reviewer, We have corrected English grammar mistakes and revised that paper.

REVIEWER 2

Concern 1

The introduction is okay but lacks application of CNN and bioinformatics state of the art research work. Author needed to cite some latest research work to cover up this gap.

Response: We are thankful for your insightful comment. Respected reviewer, We have improved the introduction section by adding a research gap and also cited state-of-the-art research work.

Concern 2

Remove the terms authors written each time with first author name or use words like this research work.

Response: The authors are grateful for your valuable suggestions. Respected reviewer, We have corrected it.

Concern 3

Add some justification about why RF KNN and LR are ensembled.

Response: Respected reviewer, We have added the justification in the paper with the following lines:

Ensembling Random Forest (RF), K-Nearest Neighbors (KNN), and Logistic Regression (LR) models offer a strategic advantage by combining their diverse learning approaches to improve predictive performance. This ensemble aims to leverage the strengths of each model: RF's robustness, KNN's pattern recognition, and LR's probabilistic interpretation, thereby enhancing overall accuracy, reducing overfitting, improving robustness against outliers, and providing a more comprehensive analysis of the cervical cancer dataset. The ensemble, facilitated by a voting classifier, fosters a collective decision-making process, resulting in a more robust and accurate predictive model for cervical cancer detection.

Concern 4

Add an example to show how the voting classifier works for ensemble learning.

Response: The authors highly appreciate your valuable suggestions. Respected reviewer, we have added the algorithm with example in the paper at page 7 and 8 in the paper.

Concern 5

Explain with reference, what is class imbalance problem and why it is necessary to solve

Response: Respected reviewer, WE have added an explanation in the paper with the following lines:

Class imbalance in datasets emerges when one group considerably outnumbers others, providing issues for ML models by biassing them towards the majority class [2]. This imbalance can lead to worse detection of minority groups, for as when identifying illnesses like cancer, affecting model accuracy and patient outcomes. Addressing this imbalance is vital for ensuring fair and accurate learning, eliminating biases towards majority classes, and boosting the model's capacity to recognise all classes successfully, especially in critical areas like medical diagnosis.

Concern 6

Why Adasyn features, why not smote?

Response: Respected reviewer, ADASYN (Adaptive Synthetic Sampling) is an improved version of SMOTE (Synthetic Minority Over-sampling Technique) [3]. ADASYN is preferred over SMOTE due to its adaptive nature, focusing on generating synthetic samples for challenging minority class instances. ADASYN's adaptability near the decision boundary helps handle data complexities and aims to improve model performance by addressing harder-to-learn instances within the minority class. This approach potentially leads to a more refined oversampling strategy, especially beneficial in scenarios like medical diagnosis where capturing nuanced variations is crucial for accurate predictions. ADASYn is being used in disease diagnosis to handle class imbalance [1].

Concern 7

English editing is compulsory.

Response: We are thankful for your insightful comment. Respected reviewer, We have corrected English grammar mistakes and revised that paper.

References

[1] Gulnaz Ahmed, Meng Joo Er, Mian Muhammad Sadiq Fareed, Shahid Zikria, Saqib Mahmood, Jiao He, Muhammad Asad, Syeda Fizzah Jilani, and Muhammad Aslam. Dad-net: Classification of alzheimer's disease using adasyn oversampling technique and optimized neural network. *Molecules*, 27(20):7085, 2022.

[2] Erendira Rendon, Roberto Alejo, Carlos Castorena, Frank J Isidro-Ortega, and Everardo E Granda-Gutierrez. Data sampling methods to deal with the big data multi-class imbalance problem. *Applied Sciences*, 10(4):1276, 2020.

[3] Venkata Pavan Kumar Turlapati and Manas Ranjan Prusty. Outlier-smote: A refined oversampling technique for improved detection of covid-19. *Intelligence-based medicine*, 3:100023, 2020.
